# Annexin A2 Mediates Dysferlin Accumulation and Muscle Cell Membrane Repair

**DOI:** 10.3390/cells9091919

**Published:** 2020-08-19

**Authors:** Daniel C. Bittel, Goutam Chandra, Laxmi M. S. Tirunagri, Arun B. Deora, Sushma Medikayala, Luana Scheffer, Aurelia Defour, Jyoti K. Jaiswal

**Affiliations:** 1 Center for Genetic Medicine Research, 111 Michigan Av NW, Children’s National Hospital, Washington, DC 20010, USA; dbittel@childrensnational.org (D.C.B.); gchandra31@gmail.com (G.C.); doc_sushma33@yahoo.com (S.M.); LScheffer@BTFILOH.org (L.S.); defouraurelia@hotmail.fr (A.D.); 2 Department of Cellular Biophysics, The Rockefeller University, New York, NY 10065, USA; mtirunagar@mail.rockefeller.edu; 3 Department of Cell & Developmental Biology, Weill Cornell Medical College, New York, NY 10065, USA; adeora@med.cornell.edu; 4 Department of Genomics and Precision medicine, George Washington University School of Medicine and Health Sciences, Washington, DC 20010, USA

**Keywords:** muscle injury, plasma membrane, vesicle, muscular dystrophy

## Abstract

Muscle cell plasma membrane is frequently damaged by mechanical activity, and its repair requires the membrane protein dysferlin. We previously identified that, similar to dysferlin deficit, lack of annexin A2 (AnxA2) also impairs repair of skeletal myofibers. Here, we have studied the mechanism of AnxA2-mediated muscle cell membrane repair in cultured muscle cells. We find that injury-triggered increase in cytosolic calcium causes AnxA2 to bind dysferlin and accumulate on dysferlin-containing vesicles as well as with dysferlin at the site of membrane injury. AnxA2 accumulates on the injured plasma membrane in cholesterol-rich lipid microdomains and requires Src kinase activity and the presence of cholesterol. Lack of AnxA2 and its failure to translocate to the plasma membrane, both prevent calcium-triggered dysferlin translocation to the plasma membrane and compromise repair of the injured plasma membrane. Our studies identify that Anx2 senses calcium increase and injury-triggered change in plasma membrane cholesterol to facilitate dysferlin delivery and repair of the injured plasma membrane.

## 1. Introduction

Mechanical strain associated with muscle contraction routinely damages the myofiber plasma membrane (PM) [1]. For continued functioning of muscle tissue in the face of persistent myofiber PM injury, myofibers need to efficiently repair these injuries [2]. This is achieved with the help of the muscle membrane protein dysferlin, mutations in which impair myofiber repair leading to muscle degeneration [3,4,5]. Dysferlin is a member of the Ferlin protein family that contains calcium and membrane binding FerA and C2 domains [6]. Ferlin proteins are similar to the C2 domain-containing proteins such as synaptotagmins, which facilitate Ca^2+^-triggered vesicle fusion [6,7]. Thus, dysferlin is implicated in facilitating injury-triggered membrane fusion to enable plasma membrane repair (PMR) [8]. Studies from invertebrate egg cells and cultured mammalian cells have identified a role of membrane fusion in repairing the injured PM [9]. Prior studies show that dysferlin and associated PMR proteins accumulate at the site of sarcolemma damage in mature myofibers [10,11]. Using dysferlin deficient mouse myofibers and patient myoblasts, we have identified that dysferlin helps tether lysosomes at the PM, which allows PMR by enabling rapid lysosome fusion following PM injury [12]. Dysferlin localizes to cell and internal membranes in uninjured cells and is enriched in cholesterol-rich PM domains that are internalized upon injury [13,14,15,16] PM injury also results in injury-triggered dysferlin accumulation at the repairing PM, but the existing PM repair mechanisms fail to fully explain this injury-triggered increase in PM dysferlin or if this accumulation plays a role in PMR [12,13,14,15,16,17,18,19,20]. 

In recent years, annexins A1, A2, A4, A5, and A6 have been recognized for their contribution to PMR in myofibers and in other cells [11,15,19,21,22,23,24,25,26,27]. Annexins interact with membrane phospholipids in a calcium-dependent manner and serve as sensors linking Ca^2+^ signals with changes in the membrane that they bind [24,28]. Annexins affect PMR through numerous mechanisms—promoting membrane blebbing and shedding [11,22,29,30], increasing membrane curvature and closure [22], assembling membrane reinforcing protein arrays [26], and interacting with the cytoskeleton to stabilize the wounded membrane [25,31]. Annexins also facilitate calcium-triggered aggregation and fusion of membranes, processes that have been suggested to regulate PMR [15,27,32]. In response to myofiber PM injury, annexins accumulate at the site of repair [11,15]. While annexins A2 and A6 are required for skeletal myofiber PMR, AnxA1 is dispensable in mammalian skeletal myofiber repair [19,23,33]. Use of mice lacking AnxA2 shows the requirement of AnxA2 for repairing injured skeletal myofibers in a manner that is synergistic with dysferlin-mediated PMR [23]. Further, AnxA2 has been shown to directly interact with dysferlin in myotubes, which is suggested to be mediated by the N-terminal C2 domains of dysferlin [34,35,36].

Here, we examine the mechanism by which AnxA2 interacts with dysferlin and if this mediates myoblast PMR. We have employed live imaging and biochemical analysis to study the response of AnxA2 and dysferlin to PM injury and monitor their accumulation on the cell and the intracellular membranes. Live cell imaging showed that within seconds of PM injury-triggered calcium increase, AnxA2 accumulates on dysferlin vesicles and the injured PM, accumulating at the site of PMR. Accumulation of AnxA2 at the injured PM requires cholesterol and Src kinase activity. AnxA2 knockdown or inhibition of its PM translocation prevents PM accumulation of dysferlin, and compromises PMR. These studies point to a requirement of AnxA2 for the repair of PM injury and for the injury-triggered exocytic delivery of dysferlin to the PM.

## 2. Methods

### 2.1. Cell Culture and Treatment

C2C12 myoblast cell line was maintained in high glucose Dulbecco’s modified Eagle’s medium (DMEM) with Glutamax and sodium pyruvate (Invitrogen) supplemented with 10% fetal bovine serum (Hyclone, Thermo Fisher Scientific, Waltham MA, USA) and 100 µg/mL penicillin and streptomycin (Invitrogen, Carlsbad, CA, USA). For live cell imaging of proteins, cultured myoblasts were plated on 25 mm (#1.5) glass coverslips (at ~4000 cells per cm^2^), and after growing overnight, plasmids were transfected using Lipofectamine LTX (Life Technologies, Carlsbad, CA, USA). Plasmids utilized include dysferlin-GFP, caveolin-1 RFP, annexin A2 mCherry and annexin A2-GFP (1–2 μg per ~40,000 cells). 

For differentiation into myotubes, annexin A2-GFP-transfected cells were grown to confluence and then put for 3 days in differentiation media (low-glucose DMEM (1 g/L) (GIBCO/Invitrogen), supplemented with 5% horse serum (Thermo Fisher #16050-122) and 1% penicillin/streptomycin antibiotic. Three days after commencement of differentiation, transfected myotubes were assessed for annexin A2 repair response as described below (see Section 2.2.1—Laser Injury). For AnxA2 knockdown, C2C12 cells were transfected with pSuper empty vector (control) or with AnxA2 shDNA. Individual clones were selected using 400 mg/mL G418 (Thermo Fisher) and used for further analysis. 

#### Generating Immortalized Annexin A2 Knockout Myoblasts for Laser Injury Assays

AnxA2-KO mice were crossed with H-2K^b^-tsA58 immortomouse [37] (The Jackson laboratory, Bar Harbor, ME, USA) to produce homozygous mice lacking the AnxA2 gene and with the SV40 large T antigen transgene. AnxA2 KO was confirmed using genomic DNA PCR as described previously [23]. Presence of SV40 large T carrier transgene was confirmed using PCR (forward primer: T ant-R 5′ GAG TTT CAT CCT GAT AAA GGA GG. Reverse primer: T ant-F 5′ GTG GTG TAA ATA GCA AAG CAA GC). EDL muscles were harvested from two-week-old mice and placed in DMEM media containing fresh collagenase (2 mg/mL) in a 35 °C shaking water bath for under 2 h and dissociated into isolated fibers via trituration. Viable, isolated fibers were chosen and plated individually and cultured at 33 °C at 10% of CO_2_ in a 24-well plate coated with Matrigel at 1 mg/mL in DMEM +20% FBS +2% L-glutamine +2% *v*/*v* chicken embryo extract +1% penicillin and streptomycin. Media was supplemented with fresh gamma-interferon at 20 U/mL (added every two days). Fibers were removed as individual myoblasts or clones were visible. These clones were allowed to proliferate to 40% confluence, were harvested and expanded independently into clonal cultures. These conditionally immortalized AnxA2 knockout and control C57bl6 mouse myoblast clones were cultured at 33 °C due to the heat labile nature of the SV40 large T antigen, which is expressed under the control of interferon-gamma (IFN-γ). Myoblasts were cultured on gelatin-coated dishes (0.01% gelatin) until reaching ~70% confluence, at which time they were plated on glass coverslips and subjected to FM-dye repair assays described below (see Section 2.2.1—Laser Injury). 

### 2.2. Injury Assays

These were performed as reported previously [38] and described below.

#### 2.2.1. Laser Injury 

Cells cultured on coverslips were transferred to cell imaging media (CIM-HBSS with 10 mM HEPES, with (+Ca2^+^) or without added 1 mM calcium-chloride (−Ca2^+^), pH 7.4), with or without 1 mg/mL FM1-43 dye (Life Technologies). The coverslipds were placed in a microscopy stage-top ZILCS incubator (Tokai Hit Co., Fujinomiya-shi, Japan) maintained at 37 °C. For laser injury, a 1- to 5-µm^2^ area was irradiated for 10 ms with a pulsed laser (Ablate! 3i Intelligent Imaging Innovations, Inc. Denver, CO, USA). Cells were imaged using an IX81 microscope (Olympus America, Center Valley, PA, USA), in either confocal or total internal reflected fluorescence (TIRF; penetration depth = 150 nm) mode. For confocal imaging, the imaging plane was set at the membrane-coverslip interface or in the middle of the cell body. Images were acquired with a 60 × /1.45 numerical aperture oil objective and a 561-nm, and 488-nm laser (Cobolt). Kinetics of plasma membrane repair was determined via real-time tracking of cellular FM dye intensity (ΔF/F, where F is the original fluorescence intensity pre-injury) over the repair period. Membrane translocation of fluorescently-tagged repair proteins (dysferlin, annexin A2) and cholesterol lipids was determined in the same manner (ΔF/F, where F is the original fluorescence intensity of the fluorescent protein or cholesterol).

#### 2.2.2. Dysferlin Vesicle Fusion Assessment 

Tracking of dysferlin vesicle fusion was conducted as previously described [39,40]. Briefly, dysferlin-GFP transfected myoblasts (*n* = 5) were imaged using TIRF microscopy (penetration depth = 150 nm), and laser-injured as described above. 5–10 individual dysferlin-labeled vesicles were tracked over the repair/resealing period per cell to obtain the following parameters—total fluorescence emission intensity, peak/maximal fluorescence intensity, and the width^2^ of its intensity profile (in μm^2^) assessed at each timepoint post-injury for each vesicle (via SlideBook image analysis software—3i Intelligent Imaging Innovations, Inc. Denver, CO, USA). The generated fluorescence kinetics and size characteristics curves for each dysferlin vesicle were averaged with all other vesicles analyzed, to obtain an average trace of dysferlin vesicle dynamics upon membrane injury. From these parameters, vesicle fusion was established using the following criteria—1. total and peak fluorescence curves must increase rapidly; 2. total fluorescence intensity should remain elevated (as the fluorophores from the vesicle are delivered to the plasma membrane) while the peak fluorescence intensity decreases (due to the lateral spread of fluorphores within the cell membrane); 3. fluorophores spread in the plasma membrane at a rate that is comparable to the diffusion coefficient of the dysferlin-GFP protein [39,40].

#### 2.2.3. Kymograph Analyses

Co-transfected myoblasts (annexin A2-GFP+ dysferlin mCherry; annexin A2-GFP+caveolin-1-RFP) were cultured and subjected to laser injury as described above. Images were captured at two-second intervals, with 488-nm and 561-nm lasers (exposure = 100 ms each), over a 25–30 s timeframe. Post-processing for kymograph analyses was performed with Slidebook software. To obtain kymographs, a line was drawn (5–10 µm length) across the cell depicting potential colocalization of repair-associated proteins (annexin A2+dysferlin, annexin-A2+caveolin-1) from the time-lapse videos. The fluorescence signature of the drawn line, for both fluorescent channels (each for a different repair-associated protein) was plotted in-sequence over the entire capture period onto a two-dimensional kymograph (y-axis = time, x-axis = sampling line distance). Kymograph sampling was initiated prior to laser injury and concluded ~20 s after injury to track temporal changes in protein co-localization. 

#### 2.2.4. Glass Bead Injury

Transfected cells cultured on coverslips were transferred to CIM alone (injury-induced protein translocation experiments) or CIM+ 2 mg/mL of lysine-fixable FITC-dextran (Life Technologies) (repair capacity experiments). Cells were injured by rolling glass beads (Sigma-Aldrich) over the cells, allowed to heal at 37 °C for 5 min, and then either 1. immediately fixed (4% PFA, injury-induced protein translocation experiments), or 2. incubated at 37 °C for 5 min in CIM/PBS buffer +2 mg/mL of lysine-fixable TRITC dextran (Life Technologies), followed by Paraformaldehyde (PFA) fixation (repair capacity experiments). Cell nuclei were counterstained with Hoechst 33,342, and cells were then mounted in fluorescence mounting medium (Dako) and imaged via confocal microscopy (60 × /1.45 NA oil objective – injury-induced protein translocation experiments/20× objective – repair capacity experiments). The number of FITC-positive cells (injured and repaired) and TRITC-positive cells (injured and not repaired) were counted and expressed as a percentage of the total injured cells. 

### 2.3. Cell Membrane Cholesterol Response Assays 

To label cell membrane outer leaflet cholesterol, C2C12 cells were incubated in CIM supplemented with 5 uM Polyethylene (PEG) conjugated FITC-cholesterol (Nanocs, Inc.) and subsequently subjected to laser injury as described above. Localized cholesterol accumulation was assessed with SlideBook image analysis software (3i, Denver, CO, USA), with FITC-labelled cholesterol fluorescence intensity assessed near the injury site (ΔF/F, where F is the original FITC cholesterol fluorescence intensity value prior to injury). 

### 2.4. Cholesterol Depletion Assay

Annexin-2 transfected myoblasts were either untreated or depleted of cholesterol via 20 mM methyl-β-cyclodextrin (MβCD) treatment for 20 min at 37 °C. Untreated and cholesterol-depleted cells were subsequently imaged in CIM with or without MβCD respectively. Cells from both conditions were subsequently exposed to laser injury or 10 uM ionomycin and imaged via real-time TIRF microscopy (ionomycin experiments) or confocal microscopy (laser injury experiments) at two-second intervals to track annexin-2 cell surface translocation over a 5–15-min period. Twenty to thirty cells were assessed for annexin-2 translocation kinetics in both untreated control and MβCD-treated cells.

### 2.5. Western Blotting 

Cells were lysed with RIPA buffer (Sigma-Aldrich) containing a protease inhibitor cocktail (Fisher Scientific, Waltham, MA, USA). Proteins transferred to nitrocellulose membranes were probed with the indicated antibodies against dysferlin (Novocastra, Buffalo Grove, IL, USA), Anx A2 (BD biosciences, #610068), caveolin-1 (Abcam, #ab2910), b-actin (Cell Signaling, #4967), or cadherin (Cell Signaling, #4068). Primary antibodies were followed by the appropriate HRP-conjugated secondary antibodies (Sigma-Aldrich) and chemiluminescent western blotting substrate (Fisher, Waltham, MA, USA; GE Healthcare, Pittsburgh, PA, USA). The blots were then processed on Bio-Lite X-ray film (Denville Scientific, Metuchen, NJ, USA), and signals for AnxA2 and dysferlin protein bands were normalized to that of the internal control‒‒cadherin.

### 2.6. Membrane Fraction Protein Analysis 

Cells cultured at ~75–80% confluence in wells of a 6-well plate were used for cell surface protein pull down as described previously [17]. Briefly, untreated myoblasts or those treated with ionomycin, or those incubated for 30 min (37 °C CO_2_ incubator, in DMEM) with DMSO (control, 1 uL/mL) or Src kinase inhibitors herbimycin A (1 µM), or PP2 (10 µM), were washed in chilled Hanks’ balanced salt solution (HBSS; Sigma-Aldrich). Cells were then treated with 0.5 mg/mL cell-impermeant EZ-Link Sulfo-NHS-LC-Biotin (Thermo Fisher Scientific) made in cold HBSS (pH 7.4) and incubated for 30 min at 4 °C. This allowed biotinylating only the cell surface proteins accessible at the cell surface. Following a wash with cold HBSS, unreacted biotin was quenched with cold 0.1 m Tris-HCl solution (pH 7.4) for 15 min. Cells were then lysed using RIPA, and equal amounts of protein lysate were used to bind via MyOne Streptavidin C1 beads (250 uL), which were washed and put into SDS loading buffer. At the time of western blotting for membrane fraction-enriched proteins, beads and lysate samples were heated for 30 min at 50 °C, and bead supernatant (or cell lysates) was loaded on a 4–12% gradient MOPS NuPage gel. 

### 2.7. Dysferlin Immunoprecipitation (IP)

A confluent dish of myoblasts or myotubes that were untreated or injured by cell scraping were collected in ice-cold lysis buffer (50 mM Tris HCL, pH 7.5, 150 mM NaCl, 50 mM deoxy cholate, 0.2% Triton ×100, 1× protease inhibitor cocktail). Cells were lysed via alternate rounds of liquid nitrogen freezing and thawing, lysates centrifuged (14,000 rpm, 30-min.), and supernatant assessed for protein content. Separately, covalently conjugated protein A sepharose beads (polycloncal antibodies, GE healthcare) or protein G sepharose beads (monoclonal antibodies) were washed 3× with lysis buffer and re-suspended in lysis buffer and stored at 4 °C.

*Immunoprecipitation* – Cell lysates were pre-cleared via incubation with washed sepharose beads (1 h) and incubated in rabbit polyclonal anti-dysferlin antibody (NCL-hamlet, Leica, Wetzlar, Germany). Subsequently, the washed sepharose beads were added and incubated for 2 h. Proteins were eluted off the beads by addition of 2× sample buffer and loaded into a 4–10% tris-glycine SDS-PAGE gel (Invitrogen). Gels were processed as above for Western blot analysis using primary antibodies (Abcam rabbit anti-dysferlin, 1:500 dilution; BD biosciences anti-annexin A2 antibody, 1:2000 dilution; BD biosciences rabbit anti-caveolin-1, 1:2000 dilution). Blots were probed with secondary antibodies (anti-rabbit, 1:50,000 dilution; anti-mouse, 1:5000 dilution) for 1–2 h in 5% milk (TBST buffer) and stained with either ECL plus substrate (GE healthcare), femto super signal substrate (Thermo Scientific) for dysferlin and annexin blots, or ECL substrate (GE healthcare) for anti-caveolin detection. 

### 2.8. Statistical Analysis

One-way ANOVA followed by a Tukey’s HSD post-hoc test was used to determine differences in annexin A2 expression among clone populations, calcium-stimulated cell surface protein quantification measures, and to compare the proportion of myoblasts that failed to repair following injury in AnxA2 knockdown, knockout, and MβCD-treatment conditions. Repeated-measures ANOVA was used to assess for differences in cell-surface dysferlin and AnxA2 expression across ionomycin exposure timepoints between clone populations and membrane fractions following injury. For annexin 2 membrane translocation and myoblast repair kinetics (FM-dye-intensity kinetics), all generated curves were compared via mixed model ANOVA with analyses for interaction effects between the main effects of treatment condition and time or trial. In the event of significant interaction, group differences in FM dye fluorescence intensity/membrane fluorescence/eccentric force were assessed per time point via a Holm-Sidak test and Huynh-Feldt correction due to violation of sphericity. For all statistical analysis, alpha level was set at *p* < 0.05. 

## 3. Results

### 3.1. Cell Injury Triggers AnxA2 and Dysferlin Co-Accumulation on the Membrane

**Figure 1:**
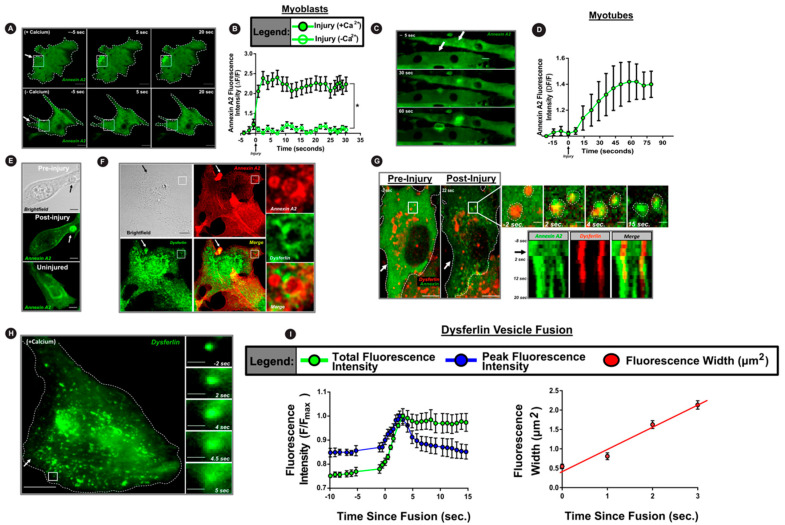
Annexin 2 and dysferlin co-accumulate at injured myoblast membrane. (**A**–**D**) Confocal images of (**A**) myoblasts and (**C**) myotubes expressing AnxA2-GFP (green) during focal laser injury (site marked by white arrow). (**B**,**D**) Plots showing the intensity of AnxA2-GFP at the site of membrane injury in myoblasts injured in the presence or absence of extracellular calcium (**B**) and in myotubes injured in the presence of extracellular calcium (**D**). (*n* = 12 cells/myotubes per condition, B: * *p* < 0.05, main effect of treatment condition). (**E**,**F**) Confocal images of glass-bead-injured myoblasts stained for (**E**) endogenous AnxA2 or (**F**) AnxA2 and dysferlin. Arrows indicate the site of glass bead injury. Inset in panel F shows vesicle in the injury-proximal region (marked by white box). (**G**) Confocal images at the membrane-coverslip interface of dysferlin-mCherry and AnxA2-GFP co-transfected myoblasts injured in the presence of calcium (arrow marks the injury site). Images in the inset are of the white box showing rapid AnxA2 translocation to dysferlin-containing vesicles prior to its fusion. Kymograph depicts the vesicles highlighted in G and spans the duration of the repair response, beginning at eight seconds prior to injury—black arrow indicates time of membrane injury. (**H**) TIRFM images of dysferlin-GFP-expressing myoblasts with the inset representing the white box and showing fusion of a single dysferlin-containing vesicle following injury (white arrow). Scale bar = 10 um. (**I**) Plots showing averaged fluorescence intensity of dysferlin-GFP vesicles imaged using TIRFM. Green trace depicts total fluorescence intensity. Blue trace shows peak fluorescence intensity. Red trace shows the area (width^2^) of the dysferlin-GFP fluorescence over the course of vesicle fusion and beyond. Traces are the average of *n* = 5 cells (5–10 dysferlin vesicles per cell). All data are presented as mean ± SEM. * *p* < 0.05 vs. -calcium condition determined via mixed model ANOVA with analyses for interaction effects between treatment condition and time. Scale bar = 10 µm (insets = 1 um).

Annexins are cytosolic proteins that accumulate on the injured plasma membrane. As injury-triggered calcium influx is a major stimulus for annexin response to PM injury, we examined injury-triggered and Ca^2+^-dependent PM translocation AnxA2 in the muscle cell. Confocal microscopy of myoblasts expressing GFP-tagged AnxA2 showed that within seconds of injury, AnxA2 accumulates at the site of PM injury (Figure 1A,B and Video S1). Next, we examined this response of AnxA2 in differentiated myotubes and found AnxA2 responded similarly by accumulating at the site of PM injury (Figure 1C,D). Injury-triggered accumulation of AnxA2 required calcium influx, as cells injured in the absence of extracellular calcium showed no AnxA2 translocation at the injury site (Figure 1A,B). This response of AnxA2 occurred independently of the mode of PM injury, as PM injury by glass beads resulted in accumulation of the endogenous AnxA2 at the site of injury (Figure 1E). Interestingly, at the site of injury, AnxA2 co-accumulated with the transmembrane protein dysferlin (Figure 1F). Moreover, we observed that in these injured cells, AnxA2 also accumulated on dysferlin-containing intracellular vesicles (Figure 1F inset). To visualize this process in real time, we used live cell spinning disc confocal imaging together with focal laser injury to monitor the response of GFP-tagged AnxA2 in myoblast expressing mCherry-tagged dysferlin. Within seconds of being injured, cytosolic AnxA2 rapidly translocated to membrane-proximal dysferlin-containing vesicles (Figure 1G, inset, kymograph and Video S2). Independently, to assess the fate of the dysferlin-containing vesicles near the plasma membrane upon focal laser injury, we employed total internal reflection fluorescence microscopy (TIRFM). PM injury caused the dysferlin-containing vesicles to rapidly disappear (Video S3) with injury causing the vesicles to release dysferlin-GFP (Figure 1H, inset, Video S4). To assess if this is due to vesicle fusion or injury-triggered vesicle movement we used the approach that we have previously described to assess the fusogenic fate of vesicles using TIRFM (see Methods). This analysis identified that injury resulted in rapid delivery of the dysferlin-GFP protein from the vesicle to the PM, and in the PM, the protein diffused away from the site of fusion at a rate of 0.6 µm^2^/s, a rate consistent with the diffusion of membrane proteins (Figure 1I). Together, these results demonstrate that Ca^2+^ influx following focal PM muscle injury in myoblasts triggers rapid accumulation of AnxA2 and dysferlin at the injury site which is co-incident with fusion of dysferlin-containing vesicles.

### 3.2. PM Translocation of AnxA2 Enables Ca^2+^-Triggered PM Accumulation of Dysferlin

**Figure 2:**
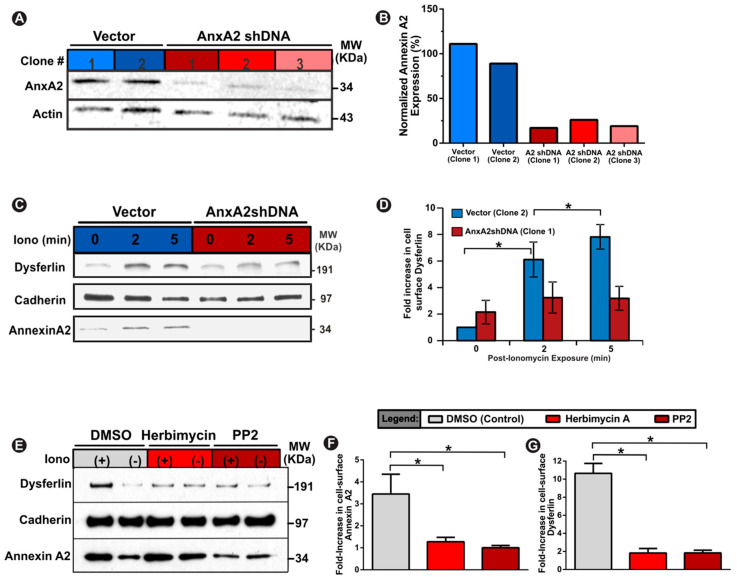
Annexin A2 enables Ca^2+^-dependent increase in cell surface dysferlin. (**A**,**B**) Western blot images (**A**) and quantification (**B**) of control vector clones and Annexin A2 knockdown clones demonstrating ≤ 20% annexin A2 expression. (**C**) Western blot images and (**D**) quantification of cell-surface dysferlin in vector and annexin 2 knockdown cells at specified timepoints after calcium stimulation with calcium ionophore. (**E**) Western blot images and (**F**,**G**) quantification of cell-surface dysferlin and annexin 2 in ionomycin-treated cells co-treated with either DMSO (control) or annexin 2 phosphorylation inhibitors (herbimcyin A and PP2). Fold-increase values represent intensity of specific protein band normalized to respective cadherin protein band. Data is presented as mean ± SEM, *n* = 3 biological replicates. * *p* < 0.05 vs. Vector Clone 2 (**B**,**D**) or DMSO (**F**,**G**). (**B**,**F**,**G**) assessed via one-way ANOVA, (D) assessed via repeated measures ANOVA, alpha set at *p* < 0.05.

With injury in the presence of calcium triggering AnxA2 and dysferlin co-accumulation at the injury site and on dysferlin-containing vesicles, which fuse with the injured membrane, we examined if AnxA2 facilitates calcium-triggered PM accumulation of dysferlin. To test this, we generated C2C12 myoblast cell lines stably knocked down (>80%) for AnxA2 (Figure 2A,B). To mimic large cytosolic Ca^2+^ increase (without PM damage) we treated cells with calcium ionophore, which acutely increases the cytosolic Ca^2+^ while keeping the plasma membrane intact. This approach allows selective biotinylation of cell membrane proteins that are detectable extracellularly using the approach we previously described [17,18]. AnxA2 and dysferlin showed a time-dependent increase in their cell surface levels in control (empty vector) myoblasts, but this was prevented in AnxA2 knockdown myoblasts (AnxA2shDNA) (Figure 2C,D). We next examined if Ca^2+^-triggered cell surface translocation of dysferlin required just the presence of AnxA2 or if it also required translocation of AnxA2 to the PM. Previous studies have identified the requirement of tyrosine 23 phosphorylation of AnxA2 by Src kinase for its PM trafficking [41,42]. Thus, to block AnxA2 phosphorylation we treated healthy myoblasts with two independent Src kinase inhibitors—herbimycin-A and PP2—which effectively impair phosphorylation of tyrosine 23 residue of AnxA2 and prevent its membrane translocation [41]. Following an acute (20 min) treatment with either one of these inhibitors, we found that Ca^2+^ increase failed to trigger PM translocation of AnxA2, monitored using a cell surface biotinylation approach (Figure 2E–G). Concomitantly, this also reduced Ca^2+^-triggered PM accumulation of dysferlin by ~10-fold (Figure 2E–G). Together, the above findings highlight that AnxA2 facilitates Ca^2+^-triggered translocation of dysferlin to the injured PM. 

### 3.3. Cholesterol is Required for AnxA2 and Dysferlin Accumulation at the Injured PM

**Figure 3:**
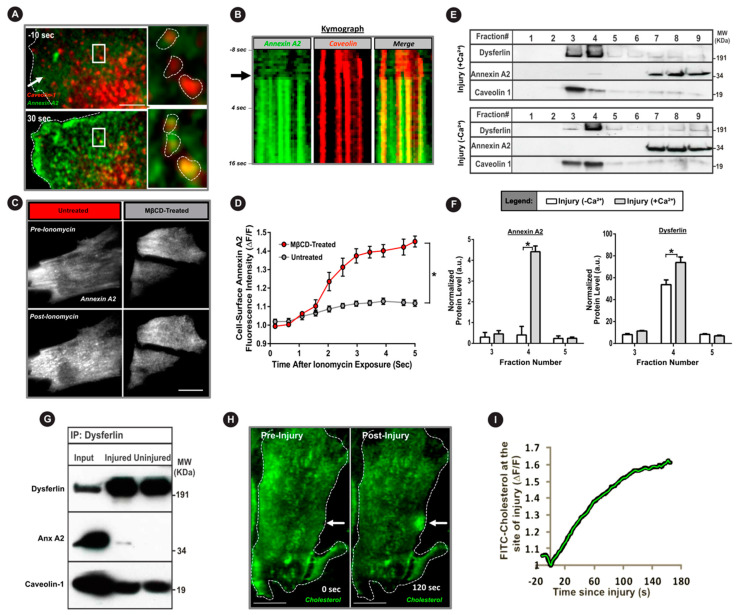
Injury causes binding of annexin A2 with dysferlin and co-accumulation in cholesterol-rich membrane domains. (**A**) TIRFM images of myoblasts co-expressing caveolin-1-RFP (red) and AnxA2-GFP (green) following laser injury (white arrow) in the presence of calcium. Inset shows zoom of white boxes demonstrating injury-induced translocation of AnxA2 to caveolin-enriched membrane domains. (**B**) Kymograph showing colocalization kinetics of AnxA2 (green) at the caveolin-rich membrane regions (red) following PM injury. Kymograph spans the duration of repair response beginning at eight seconds pre-injury and the black arrow indicates time of injury. (**C**) TIRFM images and (**D**) plot quantifying AnxA2 (white) membrane translocation upon calcium stimulation (ionomycin) in untreated and cholesterol-depleted (MβCD-treated) cells (*n* = 20 cells per condition, 4 biological repeats). (**E**) Western blot images and (**F**) quantification of the membrane fraction of myoblasts injured in the presence or absence of extracellular calcium (*n* = 4). (**G**) Western blot of injured and uninjured myoblasts immunoprecipitated using anti-dysferlin antibody and probed for AnxA2 and caveolin co-immunoprecipitation. (**H**,**I**) Confocal images (**H**) and quantification (**I**) of FITC-cholesterol after focal laser injury (white arrow) in a myoblast. All data are presented as mean ± SEM. * *p* < 0.05 vs. - MβCD-treated (**D**) or Ca^2+^ condition (**F**). Differences in the kinetics of cell-surface AnxA2-GFP upon ionomycin exposure (**D**), determined via mixed model ANOVA with analyses for interaction effects between treatment condition and time. Differences in membrane fraction protein quantification (**F**), determined via repeated-measures ANOVA. Scale bar = 10 µm.

Lipid microdomains facilitate PM interaction of AnxA2 and enable regulated exocytosis in lung and chromaffin cells [43,44,45]. Further, in non-muscle cells, dysferlin resides in cholesterol-rich lipid microdomains [46,47], and PM lipids (sterols and sphingomyelin) are involved in muscle cell repair [10,48]. Caveolae are the PM compartments enriched in cholesterol and sphingomyelin lipids. Dysferlin interacts with the caveolar protein caveolin, and caveolae support dysferlin endocytosis and PMR [49,50]. We thus examined the involvement of cholesterol and caveolae in AnxA2-dysferlin interaction and their injury-triggered membrane accumulation. Using live cell confocal imaging of PMR in myoblasts co-expressing AnxA2-GFP and caveolin-1-RFP, we observed rapid injury-triggered AnxA2 co-localization with caveolin-1 (Figure 3A,B, Video S5). Next, we examined if Ca^2+^-triggered PM translocation of AnxA2 was cholesterol dependent by using TIRF microscopy of myoblasts expressing AnxA2-GFP. Treatment with calcium ionophore caused a rapid PM translocation of AnxA2, which was nearly abolished by acute cholesterol extraction (20 min MβCD treatment) (Figure 3C,D). To independently establish that injury-triggered increase in Ca^2+^ leads endogenous AnxA2 and dysferlin to interact in cholesterol-rich PM lipid microdomain, we injured C2C12 myotubes in the presence and absence of extracellular Ca^2+^. Following scrape injury of these muscle cells, lipid microdomains were isolated by bicarbonate extraction of the membrane followed by density gradient fractionation. Western blot analysis of the resulting fraction showed that even in the muscle cells, the majority (60–80%) of dysferlin is present in caveolin-1-containing lipid rafts (fractions 3 and 4), which are devoid of any AnxA2 when PM is injured in the absence of extracellular Ca^2+^ (Figure 3E,F). However, injury in the presence of extracellular Ca^2+^ caused AnxA2 (~5% of the total) to be localized in the same fraction with dysferlin and caveolin, and this AnxA2 presence also increased the relative amount of dysferlin in this fraction (Figure 3E,F). These results demonstrate that membrane injury induces translocation of Anx2 protein to membrane cholesterol-rich domains associated with caveolin and dysferlin protein. Further, in support of this injury-triggered AnxA2-dysferlin interaction we immunoprecipitated dysferlin in the injured and uninjured muscle cells and found that dysferlin interacts with and pulls down AnxA2 in the injured but not in the uninjured cells (Figure 3G). As cholesterol is a critical component of caveolae, we directly monitored the response of cholesterol lipids to PM injury. For this we labeled the cell membrane with fluorescent PEG-cholesterol and monitored the effect of focal injury. Similarly to AnxA2-GFP, we observed that PM injury caused the cholesterol to accumulate at the site of injury (Figure 3H,I, Video S6). These results indicate that both AnxA2 and cholesterol lipid respond to PM injury by accumulating at the site of injury and they partition there together with dysferlin in lipid microdomains.

### 3.4. AnxA2 and Its Interaction with Dysferlin is Required for Plasma Membrane Repair

**Figure 4:**
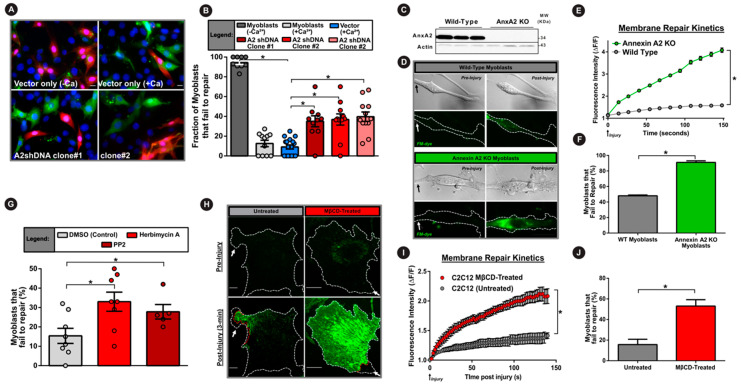
Annexin A2 and its PM translocation are required for myoblast cell membrane repair. (**A**) Images of myoblasts subjected to glass bead injury in the presence of FITC dextran (green) followed by TRITC dextran (red) to mark cells that failed to repair. (**B**) Quantification of the proportion of injured myoblasts that fail to repair (300 cells per condition, *n* = 3). (**C**) Western blot demonstrating presence or complete lack of AnxA2 protein in primary myoblasts isolated from wild-type and AnxA2 knockout mice respectively. (**D**) Brightfield and confocal images of FM dye (green) fluorescence in primary myoblasts prior to or following laser injury. (**E**) Plot showing the kinetics of intracellular FM dye fluorescence intensity change during PM repair in wild-type (green) and AnxA2-knockout myoblasts (gray) (*n* = 12 cells per condition). (**F**) Plot quantifying of the proportion of primary myoblasts that fail to repair following laser membrane injury (60–70 cells per condition, *n* = 4). (**G**) Plot demonstrating the proportion of myoblasts that fail to repair from glass bead injury (as for A,B) following Src tyrosine kinase inhibition with herbimycin A or PP2 (200 cells per condition, *n* = 5). (**H**) Confocal images of untreated (left) or cholesterol-depleted (right) myoblasts pre- and 3 min. post injury (site marked by white arrow) in the presence of extracellular FM dye (green). (**I**) Plot showing the averaged kinetics of FM-dye entry in untreated and cholesterol-depleted cells (*n* = 30 cells each). (**J**) Plot quantifying the proportion of untreated or cholesterol-extracted C2C12 myoblasts that fail to repair (30 cells per condition, *n* = 3). Data is presented as mean ± SEM. * *p* < 0.05 vs. vector-control cells (**B**), wild-type cells (**E**,**F**), DMSO-treated cells (**G**), and MβCD-treated cells (**I**,**J**). Treatment induced differences in myoblast repair was assessed via one-way ANOVA (B,G) or an independent samples t-test (**F**,**J**). For kinetics analysis (**E**,**I**) mixed model ANOVA with analyses for interaction effects between treatment condition and time was used (* *p* < 0.05, main effect of condition). Scale bar = 10 µm.

The results above identified the requirement of AnxA2, Src kinase activity and PM cholesterol in Ca^2+^-triggered dysferlin accumulation at the injured PM. We next assessed the requirement of these regulators in muscle cell PM repair. First, we used glass bead injury assay to examine the PMR ability of AnxA2 knockdown myoblast cell lines. Cells were injured in the absence (−Ca^2+^) or presence (+Ca^2+^) of extracellular calcium, which showed that compared to control cells that failed to repair in the absence of Ca^2+^, injury in the presence of Ca^2+^ led to a 10-fold improvement in PMR ability (Figure 4A,B). However, knockdown of AnxA2 caused a 4-fold increase in the number of injured myoblasts that failed to repair (Figure 4A,B). Next, to examine if AnxA2 knockout has a similar effect on myoblast repair we isolated primary myoblasts from annexin A2 knockout and wild-type mice bred with the immortomouse background (see Methods). We first established a lack of any detectable AnxA2 protein in these knockout myoblasts (Figure 4C). We then used the laser-injury assay to study their PMR and found that AnxA2 knockout significantly impaired PMR ability, with nearly all of the AnxA2 KO cells failing to repair (Figure 4D–F). Subsequently, we examined if it is the lack of AnxA2 or inhibition of PM translocation of AnxA2 and dysferlin that impairs PMR. For this we acutely treated cells with the Src inhibitors herbimycin A and PP2, as noted above. Both these inhibitors caused a two-fold reduction in the ability of myoblasts to repair from glass bead injury (Figure 4G). Lastly, we examined if the inhibition of PM translocation of AnxA2 by cholesterol depletion also affects PMR. Using the laser injury assay, we monitored the PMR kinetics and ability following focal laser injury. Depleting the PM cholesterol with MβCD caused the treated cells to take up significantly greater FM-dye as early as 15 s post injury, and it continued even three minutes post injury (Figure 4H,I). Nearly 60% of MβCD-treated cells failed to repair PM injuries, establishing the requirement of PM cholesterol in facilitating PMR (Figure 4J).

## 4. Discussion

**Figure 5:**
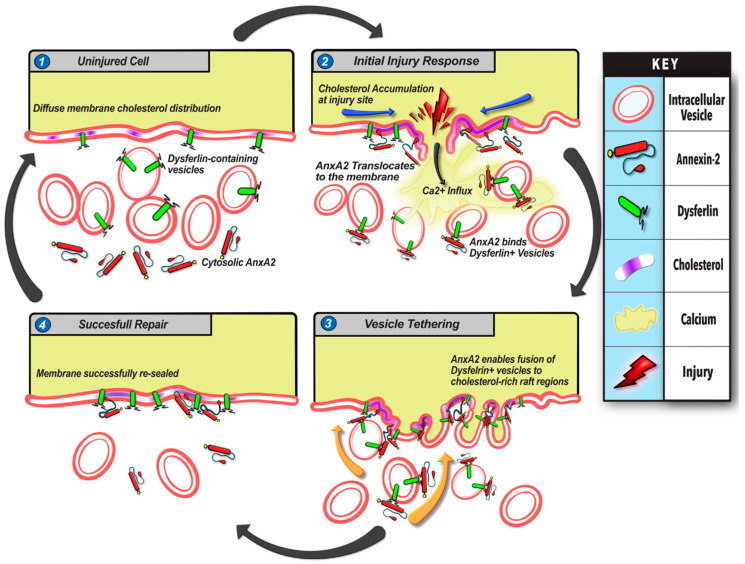
Model for annexin 2-mediated myoblast cell membrane repair. Summary of events (**1**–**4**) involved in myoblast membrane repair based on results of this study. (**1**) In the uninjured cell, AnxA2 is distributed diffusely in the cytosol, while cholesterol microdomains and associated dysferlin are distributed diffusely in the plasma membrane. Additionally, dysferlin also localizes on intracellular vesicles. (**2**) Injury to the cell membrane induces rapid influx of extracellular Ca^2+^ ions through the ruptured membrane followed by cholesterol accumulation at the injury site, AnxA2 phosphorylation, leading to its association with dysferlin and plasma membrane lipids and cholesterol-rich microdomains. (**3**) AnxA2 binding promotes fusion of dysferlin-containing vesicles with the injured membrane. (**4**) Through the cholesterol and AnxA2-mediated exocytosis of dysferlin-containing vesicles, the cell membrane level of dysferlin increases concomitantly with the repair of the wounded membrane. This allows the cell to return to a pre-injury state with redistribution of AnxA2, cholesterol, and dysferlin to their resting state. Impairment in any step of this repair pathway—reduction in AnxA2, lack of calcium influx, cholesterol depletion, or impairment of Annexin 2 phosphorylation—interferes with myoblast membrane repair.

The plasma membrane of the eukaryotic cell allows exchange and communication with the extracellular environment, while simultaneously isolating the cytoplasm from the harsh extracellular surroundings [51]. Failure to rapidly repair PM disruption results in pronounced cellular damage and cell death [51,52]. The cell’s reliance on the PMR process is even greater in mechanically active tissues such as skeletal muscle, where PM injury is frequent and failure to repair leads to degenerative diseases such as muscular dystrophies, including limb girdle muscular dystrophy 2B caused by mutations in dysferlin [3,53]. Our analysis of PMR in a muscle cell line and primary myoblasts identified that this involves PM injury-triggered responses including calcium influx, Src kinase activity, and redistribution of cholesterol, AnxA2, and dysferlin. Coordination of these events involves calcium, Src activity, and cholesterol dependent recruitment of AnxA2 following PM injury that facilitates fusion of dysferlin-containing vesicles (Figure 5). The precise choreography of ions, lipids, and proteins following injury ensures timely and efficient repair of the PM. These events are complimentary—blocking Ca^2+^ influx, Src kinase activity, or cholesterol dynamics, each blunt AnxA2 membrane translocation upon PMR. Caveolar domains in the PM serve as sites for AnxA2 binding, but they are also the site of PM-resident dysferlin, which allows synergism between Ca^2+^-triggered interaction of AnxA2 and dysferlin proteins. Injury-induced accumulation of cholesterol at the injury site enhances this further to support AnxA2 and dysferlin co-accumulation. These multipartite synergistic interactions provide a mechanism for tight spatial and temporal control of the PMR events.

Annexins are implicated in PMR through their role in facilitating membrane blebbing, shedding, and stabilization of the injured PM [11,21,22,26,29,30]. However, annexins are also suggested to regulate PMR by facilitating vesicle aggregation and fusion [15,34,54]. Indeed, the ability of AnxA2 to bind Ca^2+^ and phospholipids enables aggregation and fusion of chromaffin granules and endosomes [55,56]. In accordance with the known role of AnxA2 tyrosine phosphorylation in Ca^2+^-triggered recruitment of AnxA2 on vesicles [57], we find that injury causes AnxA2 binding to dysferlin-containing vesicles—an effect that is abrogated by the drugs that inhibit this phosphorylation (Figure 1). Subsequently, these dysferlin-containing vesicles undergo fusion (Figure 1). We show that lack of AnxA2 or use of drugs that impair its phosphorylation both impair Ca^2+^-triggered cell surface accumulation of dysferlin (Figure 2). However, with the wide range of cellular roles of Src kinase [58], future studies with AnxA2 with Tyr23-Ala mutation would help to establish the importance of this phosphorylation event in AnxA2-mediated PMR. 

At the plasma membrane, dysferlin and AnxA2 accumulate in cholesterol-rich microdomains, and lack of cholesterol impairs cell surface delivery of AnxA2 (Figure 3). Further, cholesterol interacts with AnxA2, and enhances the ability of AnxA2 to facilitate vesicle aggregation and fusion [44,45,59]. These observations implicate AnxA2 in exocytic delivery of dysferlin to the injured PM (Figure 5). However, dysferlin itself is a C2 domain-containing membrane protein implicated in regulating vesicle fusion [60]. Dysferlin deficit in muscle cells reduces rapid lysosome exocytosis and leads to the accumulation of sub-membranous vesicles [10,61]. These findings support the role of dysferlin in vesicle fusion. However, the detailed mechanism of dysferlin vesicle fusion and its regulation by AnxA2, dysferlin, or other binding partners, requires further investigation. 

Independent of the precise mechanism by which AnxA2 facilitates dysferlin accumulation at the injured cell membrane, our findings establish the importance of this process for PMR (Figure 4). These findings reinforce prior work identifying that, similar to the dysferlin deficient myofibers, AnxA2-null myofibers also show poor PMR and develop progressive myopathy, leading to muscle force loss [23]. However, AnxA2 also plays an additional role in dysferlinopathic muscle degeneration, which is due to the extracellular accumulation in the dysferlin-deficient skeletal muscles [62,63,64]. Ca^2+^ increase following cell injury triggers release of AnxA2 outside the muscle cell (Figure 2). While the extracellular role of AnxA2 in vascular physiology is well described, we have previously shown that extracellular increase in AnxA2 interferes with normal muscle tissue repair [23,64]. As a result of this, despite poor repair of AnxA2-null myofibers, they do not undergo fibroadipogenic loss [64]. Further, lack of AnxA2 in dysferlin-deficient mice protect their muscle from fibroadipogenic loss [64]. This shows AnxA2 functions in muscle both at an intracellular and extracellular level, working at the interface of muscle cell and tissue repair response. While the intracellular function of AnxA2 is to sense PM injury and contribute to coordinating a membrane repair response, AnxA2 is also secreted from the cells where it facilitates tissue-level repair response. Linking the local AnxA2-mediated membrane repair response with tissue-wide signaling is an exciting and warranted area of future studies that may likely provide insights into how cellular and tissue-level repair responses are coordinated for successful repair of muscle injuries.

## Supplementary Materials

The following are available online at https://www.mdpi.com/2073-4409/9/9/1919/s1, Video S1: Annexin A2 surface translocation with injury, Video S2: Annexin A2 transcloates to dysferlin-containing vesicles and membrane-bound dysferlin upon injury, Video S3: Dysferlin vesicle fusion upon injury, Video S4: Fusion of single dysferlin-containing vesicle upon injury, Video S5: Annexin A2 translocates to caveolin-1-rich membrane regions upon injury, Video S6: Membrane cholesterol accumulation near the site of injury.
