# Supplementary material for: Annexin A2 Mediates Dysferlin Accumulation and Muscle Cell Membrane Repair
Source: Cells. 2020 Aug 19;9(9):1919. doi: 10.3390/cells9091919 (PMC7565960; doi:10.3390/cells9091919)
Supplement: Supplementary file 1 [file cells-09-01919-s001.zip › Bittel & Chandra et al (Supplemental Material).docx]

**Supplementary Videos**

**Video 1: Injury-triggered annexin A2 accumulation at the injury site**. Time-lapse video of C2C12 myoblast expressing annexin A2-GFP, subjected to focal laser injury in CIM. Images were acquired via spinning disc confocal microscopy using a 488 nm diode laser. Cells were imaged for a period of 2 minutes. Video played at 15 Hz and time elapsed (hr:min:ss:ms format) since the start of the video is denoted in the lower right of the video panel.

**Video 2: Injury-triggered annexin A2 translocation to dysferlin-containing vesicle**. Time-lapse video showing C2C12 myoblast transfected with annexin-A2 GFP and dysferlin-mCherry. Images show the spinning-disc confocal images of the cell at the membrane-coverslip interface prior to and following injury by pulsed laser. Images were acquired using diode lasers at 488 and 560 nm for a period up to 2 minutes. Video is played at 10 Hz and time elapsed (hr:min:ss:ms format) is denoted in the upper left of the video panel.

**Video 3: Cell surface delivery of dysferlin triggered by cell membrane injury**. Time-lapse video of the response of dysferlin-GFP in C2C12 myoblast subject to focal laser injury (the lower left portion of the cell) and imaged by total-internal reflected fluorescence microscopy (TIRFM - penetration depth of 150 nm). Images collected at 2Hz and the video is played at 15 Hz. Time elapsed (hr:min:ss:ms format) since the start of the video is denoted in the lower right of the video panel. Note the loss of punctate vesicle fluorescence with the concomitant increase of the diffused membrane fluorescence.

**Video 4: Fusion of an individual dysferlin containing vesicles following cell membrane injury**. Video showing time lapse TIRF microscopy images of the response of an individual dysferlin-GFP labeled vesicle from the cell in Video 3. Dysferlin-GFP is delivered from the vesicle to the plasma membrane resulting in its diffusion of the fluorescence into the membrane. Video is acquired at 2Hz and played at 15 Hz. Time elapsed (hr:min:ss:ms format) since the start of the video is denoted in the lower right of the video panel.

**Video 5: Injury triggers accumulation of Annexin A2 on Caveolin-1 containing membrane microdomain**. Time lapse images of a C2C12 myoblast expressing Caveolin1-mCherry expressing annexin A2-GFP imaged prior to and following focal laser injury (upper left). Images show the cell at the coverslip interface imaged by spinning- disc confocal microscopy at 2Hz and video played at 10 Hz. Time elapsed (hr:min:ss:ms format) since the start of the video is denoted in the lower right of the video panel.

**Video 6: Accumulation of plasma membrane cholesterol near the site of repair**. Time-lapse video of C2C12 myoblast labeled with FITC-cholesterol and subject to pulsed laser injury. Images were acquired via spinning-disc confocal microscopy using a 488 nm laser, at the membrane-coverslip interface. Cells were imaged for a period of 3 minutes and video played at 10 Hz. Cell injured at 11.48 seconds, with injury site denoted by the green arrow.
